# Comparative Effect of Inoculation of Phosphorus-Solubilizing Bacteria and Phosphorus as Sustainable Fertilizer on Yield and Quality of Mung Bean (*Vigna radiata* L.)

**DOI:** 10.3390/plants10102079

**Published:** 2021-09-30

**Authors:** Shahid Bilal, Abu Hazafa, Imran Ashraf, Saud Alamri, Manzer H. Siddiqui, Amina Ramzan, Nimra Qamar, Farooq Sher, Muhammad Naeem

**Affiliations:** 1 Department of Agronomy, Faculty of Agriculture, University of Agriculture, Faisalabad 38000, Pakistan; Shahidbilal3240@gmail.com (S.B.); Imran.ashraf@uaf.edu.pk (I.A.); 2 Department of Biochemistry, Faculty of Sciences, University of Agriculture, Faisalabad 38000, Pakistan; 3 Department of Botany and Microbiology, College of Science, King Saud University, Riyadh 11451, Saudi Arabia; saualamri@ksu.edu.sa; 4 Department of Botany, Faculty of Sciences, University of Agriculture, Faisalabad 38000, Pakistan; aminaramzan93@gmail.com (A.R.); nimraqamar6@gmail.com (N.Q.); 5 Department of Engineering, School of Science and Technology, Nottingham Trent University, Nottingham NG11 8NS, UK; Farooq.Sher@ntu.ac.uk; 6 College of Life Science, Hebei Normal University, Shijiazhuang 050010, China; naeemsaleem413@gmail.com

**Keywords:** mung bean, phosphorus-solubilizing bacteria, grain yield, photosynthetic yield, plant productivity, *Pseudomonas*

## Abstract

Globally, the availability of phosphorus (P) to crops remains limited in two-thirds of the soils, which makes it less accessible to plants and ultimately associated with low crop yields. The present study investigated the effect of phosphorus-solubilizing bacteria (PSB; *Pseudomonas* spp.) for the improvement of phosphorus in mung bean (*Vigna radiata*) varieties and growth of net grain and biological yields. Results showed that inoculation of mung bean varieties with PSB at the rate of 100 g/kg seed significantly improved the root and shoot dry weight of about 1.13 and 12.66 g, root and shoot length of 14.49 and 50.63 cm, root and shoot phosphorus content of 2629.39 and 4138.91 mg/kg, a biological yield of 9844.41 kg/ha, number of pods of 17 per plant, number of grains of 9 per pod, grain yield of 882.23 kg/ha, and 1000-grain weight of 46.18 g after 60 days of observation. It was also observed that PSB-treated varieties of mung bean showed the maximum photosynthetic yield, photosynthetic active radiation, electron transport rate, and momentary fluorescent rate of 0.75, 364.32, 96.12, and 365.33 μmol/m^2^ s, respectively. The highest harvest index of 13.28% was recorded by P-treated mung beans. Results disclosed that inoculation of seeds of mung bean with PSB exhibited different effects in measured parameters. It is concluded that PSB possessed remarkable results in measured parameters compared to the control and highlighted that PSB could be an effective natural sustainable fertilizer for mung bean cultivation in sandy soil.

## 1. Introduction

The agriculture sector is directly or indirectly linked to 70% of the population of Pakistan, and it covers approximately 38.5% of the labor force of the country. Agriculture contributes about 18.5% to the gross national product of Pakistan [1,2]. Pulses play a significant role in Pakistan’s national economy. Although the government of Pakistan has given the agriculture sector a greater priority, the country does not have a surplus of pulses. During 2018–19, the production of pulse mash (lentil) and mung bean (*Vigna radiata*) decreased by 5.5% and 3.4%, respectively, compared to the previous year’s production [3,4].

Mung bean (*Vigna radiata* L.) is grown by marginal and resource-poor farmers mainly in the semi-arid and waterless zones in the country for grain and fodder. It contains 24% protein as a major source in the vegetarian diet and enriches soil fertility via biological nitrogen fixation [5]. It is cultured under a rainfed condition with negligible fertilizer but its production is strictly affected by water stress situations, mostly in the spring and summer, while water deficit stress leads to low grain yield [6]. It consists of 134 and 365 mg of calcium and phosphorus per 100 g of grain and contains 2–4% fats, 49.4% carbohydrates, and 25% proteins [7]. To accelerate pod maturity and seed formation in mung bean, phosphate is the most significant requirement. Phosphorus elements are not easily available in acidic soil because they are bounded by metal interactions in the soil. Phosphorus in mung beans significantly increases seed weight and pod number [7].

After nitrogen, phosphorus is the second primary macronutrient required in large amounts for plant growth. Phosphorus-solubilizing bacteria and plant growth-promoting rhizobacteria are highlighted to use phosphorus fixed in soil layers [8]. Microorganisms release different organic acids which acidify the microenvironments, and thus phosphorus solubilization takes place by replacing P ions with cations [9]. A study stated that the combined application of phosphorus-solubilizing bacterial strain and bio-organic phosphate effectively enhanced the grain yield of about 54.3% and 83.3% for Galaxy-2013 and Punjab-2011 cultivars, respectively. They also enhanced the population of phosphorus-solubilizing bacteria, organic matter, and phosphorus content in soil, resulting in a higher wheat yield under dry conditions with a limited fertilization input [10]. Phosphorus is the principal constituent in the ATP molecule that provides energy to plants for different processes such as photosynthesis, protein synthesis, translocation of nutrients, respiration, and increased root growth. It is also affected by early maturity, straw strength, crop quality, and fighting against diseases [11]. Minerals dissolution by microbes increases the minerals value in plants because 53% of total bacteria are able to convert insoluble P into soluble form. At the same time, *Burkholderiales*, *Bacillales* and genus *Pseudomonas* have greater P solubilization capability and are more abundantly present in P-poor soil than P-rich when tested in four different soils [12].

Phosphate-solubilizing bacteria take part in a starring role in P transfer and cycling to the plant to increase crop production. The use of fertilizer as a P source, as well as co-inoculation with different bacterial strains such as *Bacillus* sp. BACBR04, *Rhizobium* sp. RIZBR01, and *Bacillus* sp. BACBR06, increased shoot P content compared to uninoculated therapies receiving only soluble P [13]. *Microcystis* and *Anabaena* are phosphorus-solubilizing cyanobacteria investigated in Guanqiao ponds, which enable, by having a continuous phosphorus disponibility, the solubilization of organic and inorganic phosphorus to satisfy a wider substrate requirement. Hence, it can be determined that the PSB has a vital role in algal growth and succession. *Rhizobium* sp. of microcysts plays a positive role in the hydrolysis of carbon, nitrogen, and phosphorus [14,15]. Two varieties of mung bean, including BARI Mung-5–V1 and BARI Mung-6–V2, were tested on phosphorus fertilization effects in an agroecological area which showed a good response from V1 to increasing phosphorus fertilization of several plant pods and pod length under the use of the phosphorus treatments. It was also evaluated that both varieties demonstrate good response to the T4 treatment by comparing other treatments while maximum yield was attained from the V2T4 treatment [16].

Phosphate-solubilizing bacteria (PSB) such as *Pseudomonas striata* and *Bacillus polymyxa* revealed that growth and productivity of mung bean and fruit quality had been shown to be positive by increasing the soil microbial biomass while reducing the non-effective nodules which provide a biotechnological solution for supportable agriculture [17]. A study revealed that co-application of PSB and arbuscular mycorrhizal fungi plays a crucial role in the biogeochemical cycle like a flow of energy, improving soil acid phosphatase activities, soil organic matter decay, increasing plant biomass, and enhancing phytate mineralization [18]. An analysis found that mung bean production was improved by utilizing PSB (23 kg N/ha) and P (40 kg P/ha) in terms of the number of primary branches, the number of pods per plant, the weight of seed, and production of grains (1902.78 kg/ha) [19,20].

According to a recent situation, it is important to implement techniques that are useful for better-quality production and high yield of mung beans. Inoculation techniques with PSB are the initial step for accomplishing the objective of better quality and high mung bean yield. However, to the best of our knowledge, for the first time, the present study evaluated the potential of phosphorus-solubilizing bacteria (PSB) such as *Pseudomonas* spp. to solubilize phosphorus under phosphorus-deficient conditions and examine the role of PSB for enhancing phosphorus (P) uptake in mung beans in Pakistan. Moreover, for the first time, the present study investigated the photosynthetic yield (PSY), photosynthetic active radiation (PSAR), electron transport rate (ETR), and momentary fluorescent (MF) rate in different varieties of mung bean (V_1_ = PRI-2018, V_2_ = NM-16, V_3_ = NM-11, and V_4_ = AZRI-06) after inoculation of PSB (100 g/kg seed).

## 2. Results and Discussion

### 2.1. Agronomic Parameters Analysis

Agronomic parameters include the final emergence, fresh shoot and root weight, dry root and shoot weight, shoot and root length, number of leaves, leaf area, leaf index, and biological yield against four different varieties of mung bean with phosphorus-solubilizing bacteria (PSB) and control.

#### 2.1.1. Final Emergence

**Figure 1:**
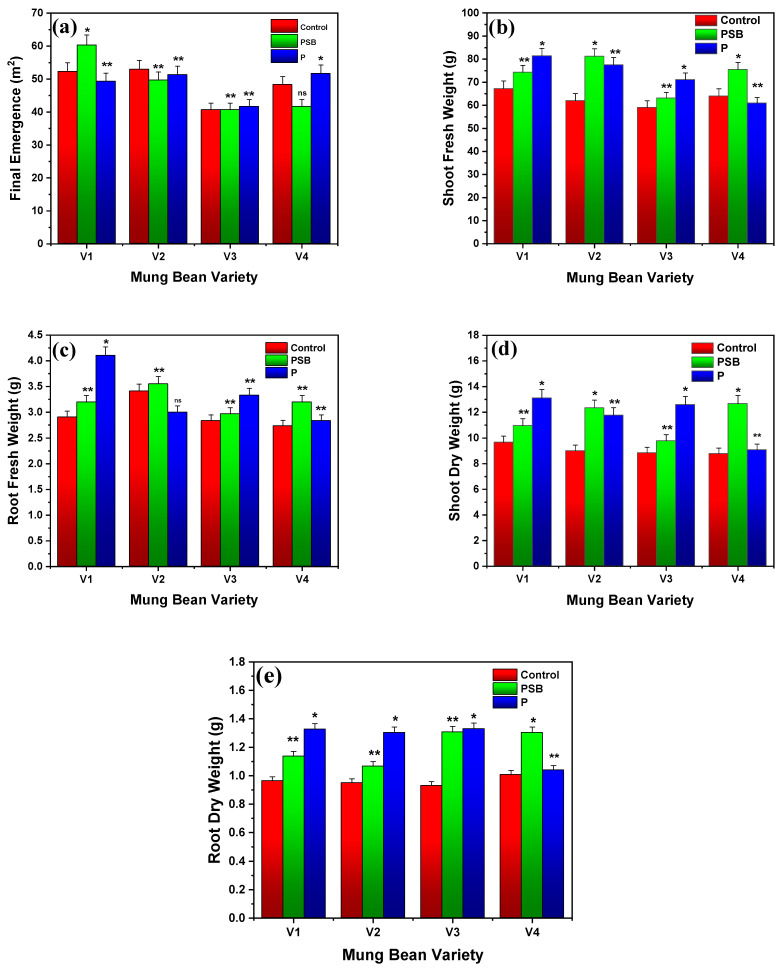
Experimental results of (**a**) final emergence, (**b**) shoot fresh weight, (**c**) root fresh weight, (**d**) shoot dry weight, and (**e**) root dry weight of different varieties of mung bean. Each treatment was measured with three replicates. The upper bar represents the standard error of the means (mean ± SEM). * Represents the highly significant (*p* ≤ 0.01), ** represents significant (*p* ≤ 0.05), and ‘ns’ represents non-significance of results at *p* ≤ 0.05.

Figure 1a shows the final emergence of four varieties of mung bean with phosphorus alone and phosphorus-solubilizing bacteria (PSB). Results indicated that the four varieties (V1, V2, V3, and V4) of mung bean showed a maximum final emergence of 52.66, 53.00, 54.33, and 48.33 m^2^ for control, 60.33, 49.66, 40.66, and 49.33 m^2^ with PSB, and 51.33, 51.66, 41.6, and 51.66 m^2^ for phosphorus alone, respectively. Results reported that PSB showed the best results in the V1 variety of mung bean compared to control and P among all varieties (*p* ≤ 0.01). The comparison between phosphorus and varieties indicated that V1 (PRI-2018) had the highest final emergence of 52.66 m^2^ and V3 (NM-11) showed the lowest emergence of 40.66 m^2^.

#### 2.1.2. Root/Shoot Fresh and Dry Weight

Figure 1b,c represents the results of fresh weight of root and shoot of four varieties of mung bean after 60 days with PSB and P-alone. By applying P-alone on fresh shoots and roots (70 kg/ha), results showed the weight of 81.43, 77.55, 71.11, and 60.88 g for a shoot, and 4.11, 3.41, 3.33, and 2.83 g for root after 60 days of measurements for V1, V2, V3, and V4, respectively. Results also indicated that PSB reported the maximum shoot and root fresh weight of about 81.22 and 3.55 g against the V2 variety, which was even greater than the control which only showed 61.98 and 3.41 g after 60 days of sowing. Outcomes disclosed that PSB showed a better fresh weight against the V2 variety of mung bean than control and P-alone (*p* ≤ 0.05).

By comparing the shoot dry weight interaction of control with P-alone (70 kg/ha), results revealed the maximum dry shoot weight of 9.66, 8.96, 8.83 and 8.77 g for control, and 13.11, 11.76, 12.58, and 9.07 g with P-alone for V1, V2, V3, and V4 of mung bean, respectively (see Figure 1d,e). At the same time, PSB showed the dry shoot weight of 10.94, 12.33, 9.77, and 12.66 g for V1, V2, V3, and V4 of mung bean, respectively. Similarly, the maximum dry root weight of 1.13, 1.32, and 0.96 g was noted for PSB, P-alone, and control, respectively, for V1 of mung bean. Overall, results reported that maximum outcomes were noted with PSB for V2 (*p* ≤ 0.01) in the case of fresh weight and V1 in the case of dry weight (*p* ≤ 0.01). Our results are correlated with the findings of Mpanga et al. [21], who examined the maximum dry shoot weight of 10 g in maize plants by applying PSM, phosphorus-solubilizing microbe (Combi-factor A) while without PSM they obtained a maximum dry shoot weight of 4 g, which proves that PSM increased the dry shoot weight. Parastesh et al. [22] reported that after 30 days of co-inoculation of wheat with vermicompost-bacterial strain (T2) significantly increased shoot dry weight and plant height of about 16.01 g/pot and 82 cm, respectively. Similarly, Khandare et al. [23] observed the maximum plant dry matter of about 10.8 and 10.5 g/plant with 75% NP + liquid inoculation (*Azotobacter*-PSB; 625 mL/ha) and 75% NP + carrier inoculation (*Azotobacter*-PSB; 10 kg/ha), respectively. Viruel et al. [24] reported that inoculated maize seed with seven strains of PSB showed a significant improvement in plant length (45%), dry weight of shoot (40%), and plant phosphorus content crop when compared to seed inoculated with a single strain of PSB.

#### 2.1.3. Numbers of Leaves, Leaf Area, and Leaf Index

Table 1 presents the number of leaves per plant for PSB- and P-alone-treated varieties of mung bean. Results showed that the maximum number of leaves of about 21, 18, and 18 per plant were observed for PSB, P-alone, and control, respectively, against V1 variety of mung bean. Similarly, V2, V3, and V4 showed the number of leaves of 17, 17, and 21 per plant for PSB, and 18, 16, and 19 per plant for P-alone. Inclusively, it is informed that PSB confirmed superior results regarding the number of leaves compared to P-alone and control. Hassan et al. [17] stated that PSB (*Pseudomonas*) showed the maximum number of leaves of 40 per plant, while PSB (*Bacillus polymyxa*) possessed the number of leaves of 41 in mung bean plants and the control showed only 11 leaves per plant.

Table 1 reveals that the maximum leaf area was recorded as 743.55 cm^−2^ with PSB in V1 (PRI-2018). Similarly, P-alone and control showed the maximum leaf area of about 628.88 and 582.22 cm^−2^ against V1 of mung bean. It is obvious from the findings that PSB presented better results regarding leaf area than P-alone and control. In addition, the highest leaf area index was observed in V1 (PRI-2018) of 2.14 cm with a phosphorus-containing sample (70 kg/ha), while PSB (100 g/kg seed) showed a maximum leaf area index of 1.77 cm only (see Table 1).

#### 2.1.4. Shoot and Root Length

**Figure 2:**
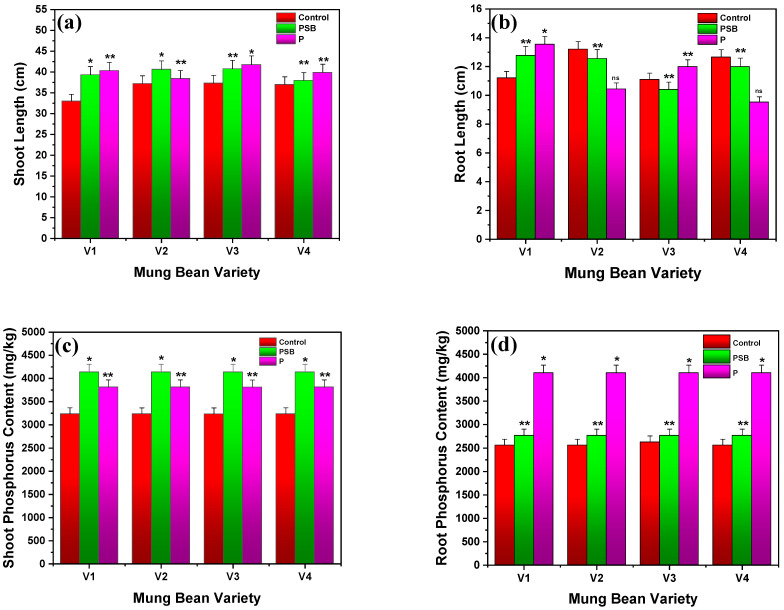
Comparative results of (**a**) shoot lengths, (**b**) root lengths, (**c**) shoot phosphorus content, and (**d**) root phosphorus content of different varieties of mung bean in inoculation with PSB and P. Each treatment was measured with three replicates. The upper bar represents the standard error of the means (mean ± SEM). * Represents *p* ≤ 0.01, ** represents *p* ≤ 0.05, and ‘ns’ represents non-significance of results.

Results revealed that the maximum shoot and root length was attained with PSB (100 g/kg seed) of about 50.63 and 14.49 cm for V2 and V1, respectively (see Figure 2). Similarly, the phosphorus-containing sample represented the maximum shoot and root length of 49.44 and 15.42 cm against V3 and V1 varieties, respectively, while the control only showed a shoot height of 44.44 cm and a root height of 12.64 cm for the same varieties after 60 days of measurement. Overall, it is concluded that the V2 variety along with PSB (100 g/kg seed) possessed the highest shoot length (*p* ≤ 0.05) (see Figure 2a). Mpanga et al. [21] reported that PSM (Combi-factor A) gives a maximum root length yield of 85.00 cm in maize plants, while without PSM, this plant shows the root length of 50.00 cm. Similarly, Rafique et al. [25] revealed the inoculation of maize plant seed with PSB (*L. fusiformis*) considerably improved the shoot and root length by about 63.1 and 44.4 cm after 45 days of harvesting. Khandare et al. [23] reported the maximum plant height of 46.3 after 60 days, and 83.85 cm after 90 days with 75% NP + liquid inoculation (*Azotobacter*-PSB; 625 mL/ha), and 46.5 cm after 60 days and 84.35 cm after 90 days with 75% NP + carrier inoculation (*Azotobacter*-PSB; 10 kg/ha), respectively.

#### 2.1.5. Root and Shoot Phosphorus Contents

Mung bean seedlings inoculated with PSB (100 g/kg seed) had shoot phosphorus contents of 4137.81, 4138.91, 4137.33, and 4135.22 mg/kg, followed by root phosphorus contents of 2562.11, 2561.33, 2629.39, and 2560.10 mg/kg, respectively (see Figure 2c,d). Mung bean variety V1 had the highest shoot and phosphorus content of 3815.86 and 4103.32 mg/kg, respectively, whereas varieties V2 and V3 had maximums of 3812.33 and 4101.22 mg/kg, respectively, and variety V4 had maximums of 3814.65 and 4104.33 mg/kg. Meanwhile, the control had shoot and root phosphorus contents of 3236.21 and 2562.10 mg/kg for V1, 3236.21 and 2562.10 mg/kg for V2, 3236.21 and 2562.10 mg/kg for V3, and 3236.21 and 2562.10 mg/kg for V4, respectively. It is observed that PSB showed remarkable shoot phosphorus content in all varieties of mung bean as compared to the control and P (*p* ≤ 0.01) (see Figure 2c), while P-treated mung bean (*p* ≤ 0.01) presented better results in root phosphorus content compared to the control and PSB (see Figure 2d). Rafique et al. [25] demonstrated that co-inoculation of maize plant seed with PSB (*L. fusiformis*) and bagasse biochar showed the N, P, and K concentrations of 3.03 ± 0.15, 0.77 ± 0.07, and 2.08 ± 0.11%, respectively, after 45 days of harvesting and 3.09 ± 0.15, 0.79 ± 0.07, and 2.18 ± 0.16%, respectively, after 65 days of harvesting.

#### 2.1.6. Biological Yield

**Figure 3:**
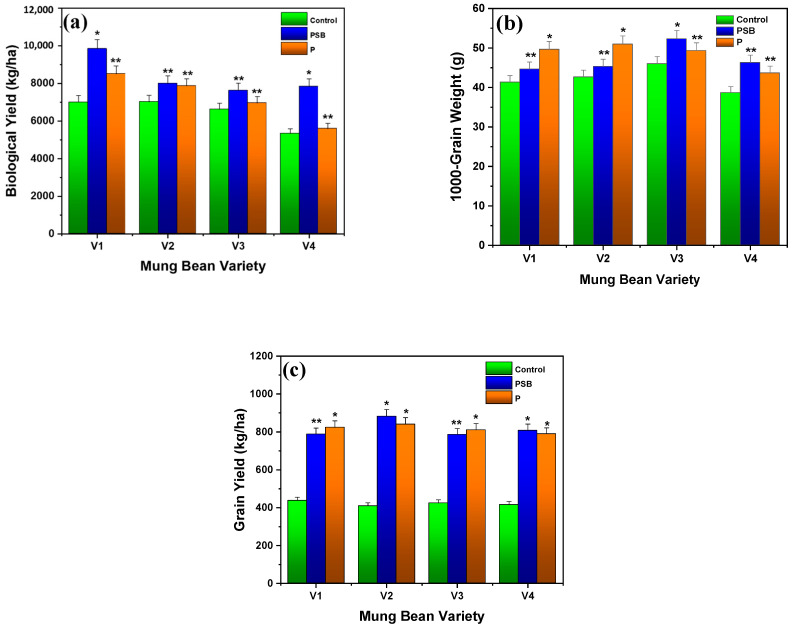
Experimental results of (**a**) biological yield, (**b**) 1000-grain weight, and (**c**) grain yield of different varieties of mung bean in inoculation with PSB (*Pseudomonas* spp.) and P after 60 days of observation. Each treatment was measured with three replicates. The upper bar represents the standard error of the means (mean ± SEM). * Represents *p* ≤ 0.01 and ** represents *p* ≤ 0.05 of results.

Biological yield is one of the most critical parameters to signify the importance of phosphorus-solubilizing bacteria (PSB) on mung beans. Figure 3a represents the results of a biological yield of four varieties of mung bean for control, PSB, and P-alone. Results revealed that the control showed the biological yield of 7000.18, 7022.21, 6622.27, and 5333.31 kg/ha for variety V1, V2, V3, and V4, respectively. PSB (100 g/kg seed) and P-alone (75 kg/ha) yielded 9844.41 and 8511.21 kg/ha for V1, 8000.11 and 7866.59 kg/ha for V2, 7622.27 and 6955.51 kg/ha for V3, and 7844.44 and 5600.13 kg/ha for V4. Results showed that maximum biological yield was attained by PSB in V1 (PRI-2018) of mung bean that was also better than the control and P (*p* ≤ 0.01). As a result, V1 (PRI-2018) has a higher yield with PSB (100 g/kg seed) than the control and P-alone (see Figure 3). Our results are in agreement with the findings of Sial et al. [26], who observed that mung beans showed the highest biological yield of 2541 and 2391 kg/ha by using phosphorus (75 kg/ha) and PSB (*B. polymyxa*: 25 mL/kg), respectively. Chattha et al. [27] reported that the mung bean plant showed a maximum biological yield of 6575.00 kg/ha by using Rhizobium with PSB, while using phosphorus (50 kg/ha) and nitrogen (20 kg/ha) this plant presented a maximum biological yield of 6560.00 kg/ha.

### 2.2. Yield Parameters Assessment

#### 2.2.1. Grain Yield

Figure 3b,c represents the results of 1000-grain weight and grain yield for inoculation of PSB- and P-treated four different mung bean varieties. Results demonstrated that the control of V1, V2, V3, and V4 showed the 1000-grain weight of 41.32, 42.65, 46.18, and 38.66 g following grain yield of 437.77, 409.13, 424.43, and 415.51 kg/ha, respectively. It was noted that the 1000-grain weight and grain yield were increased after treating the seeds of mung bean with PSB and P. The maximum 1000-grain weight for V1, V2, V3, and V4 treated varieties with PSB (100 g/kg seed) was observed as 44.65, 45.32, 52.33, and 46.66 g followed by grain yields of 788.88, 882.23, 786.64, and 808.81 kg/ha, respectively. Similarly, 49.66, 51.32, 49.32, and 43.65 g 1000-grain weights were observed for P-alone-treated (70 kg/ha) varieties of mung bean V1, V2, V3, and V4, while grain yield was noted as 824.61, 840.97, 811.13, and 790.35 kg/ha, respectively. It could be observed that mung bean (V2) inoculation with PSB presented remarkable results compared to control and P (*p* ≤ 0.01) as presented in Figure 3c. Ram et al. [28] reported that the maximum wheat grain yield was observed as 50.9 and 52.9 per spike with phosphorus and PSB, respectively, at a 0% P rate that increased to 53.2 and 53.9 per spike at a 100% P rate. They also analyzed the maximum yield of 38.2 and 37.6 g per spike at 0% P rate for 1000-grain weight that exceeded to 39.1 and 39.1 g by implementing a 100% P rate.

Sial et al. [26] observed that mung bean plants possess the highest grain yield of 3.94 g and 1000-grain weight of 4.80 g by using phosphorus (75 kg/ha), while presenting the lowest grain yield of 2.6 g and 1000-grain weight of 1.8 g in control samples, which proves that only the use of phosphorus (75 kg/ha) is more valuable for plant grain yield and 1000-grain weight. Hassan et al. [17] examined the highest 1000-grain weight of 35.2 and 33.8 g in PSB *Bacillus polymyxa-* and *Pseudomonas*-treated seeds. Zafar et al. [29] reported that the use of PSB-inoculated seed and synthetic fertilizer increased the grain yield of 1852 kg/ha and showed the highest 1000-grain weight of 18.48 g in chickpea. Chattha et al. [27] reported that mung bean plants showed the highest grain yield of 1402.2 kg/ha and 1000-grain weight of 45.7 g by using Rhizobium along with PSB, while treating with phosphorus (50 kg/ha) and nitrogen (20 kg/ha) this plant presented the grain yield of 1389.7 kg/ha and 1000-grain weight of 45.6 g. Khandare et al. [23] demonstrated the highest grain and straw yield of about 4256 and 7319 kg/ha with 75% NP + liquid inoculation (*Azotobacter*-PSB; 625 mL/ha) and 4271 and 7348 kg/ha with 75% NP + carrier inoculation (*Azotobacter*-PSB; 10 kg/ha), respectively.

#### 2.2.2. Number of Pods, Pod-Bearing Branches, and Grains

**Figure 4:**
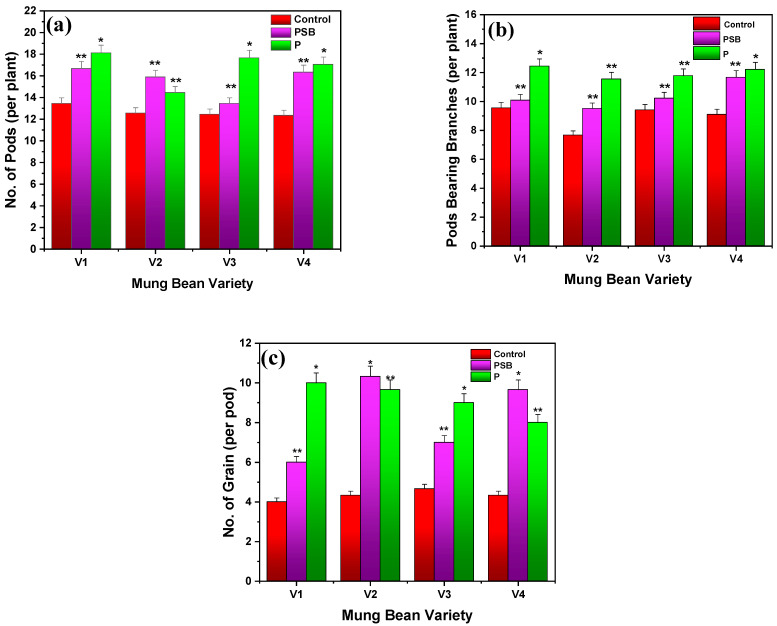
Comparative results of (**a**) number of pods, (**b**) pod-bearing branches, and (**c**) number of grains of different varieties of mung bean in inoculation with PSB and P after 60 days. Each treatment was the mean of three replicates. The upper bar represents the standard error of the means (mean ± SEM). * Represents *p* ≤ 0.01 and ** represents *p* ≤ 0.05 of results.

Figure 4a–c presents the results of the number of pods, pod-bearing branches, and grains for four different varieties of mung beans treated with PSB and P-alone. It was observed that inoculated mung beans with PSB (100 g/kg seed) showed the number of pods and pod-bearing branches of about 17 and 10 per plant for V1, 16 and 10 per plant for V2, 15 and 10 per plant for V3, and 16 and 12 per plant for V4, while the number of grains were noted to be 6, 10, 7, and 10 per pod for V1, V2, V3, and V4, respectively. Similarly, P-alone-treated mung bean revealed the maximum number of pods and pod-bearing branches of 18 and 12 per plant for V1, 14 and 11 per plant for V2, 18 and 11 per plant for V3, and 17 and 13 per plant for V4, respectively. However, the number of grains were observed as 10, 9, 9, and 11 per pod for P-treated V1, V2, V3, and V4 varieties of mung bean, respectively. Based on the results, it was noted that P-inoculated (70 kg/ha) mung bean showed a better number of pods, pod-bearing branches, and grains per pod as compared to the control followed by PSB-inoculated mung beans (*p* ≤ 0.01). Thus, it has resulted that direct use of P is more valuable for the increase in the number of pod and grains than PSB (see Figure 4). Ebbisa and Amdemariam [30] reported that faba bean plants showed the highest number of pods of about 19.43 per plant by applying biofertilizer of farmyard manure with inoculation of Rhizobium at a 150% rate of NPS, while the lowest number of pods of about 11.57 per plant was recorded by applying biofertilizer of farmyard manure without Rhizobium inoculation at 0% rate of NPS. Zafar et al. [29] experimented on chickpea and revealed that the use of PSB along with synthetic fertilizer showed the highest number of pods and number of grains of 36.66 and 1.58 per plant, respectively, while the maximum number of grains were noted as 4 per pod. Hassan et al. [17] experimentally conducted the result that inoculation of PSB increased the number of branches in the mung bean plant. They observed the highest number of branches of 39 in PSB of *Bacillus polymyxa* and *Pseudomonas*, while the control showed 19 branches per plant.

#### 2.2.3. Harvest Index

**Figure 5:**
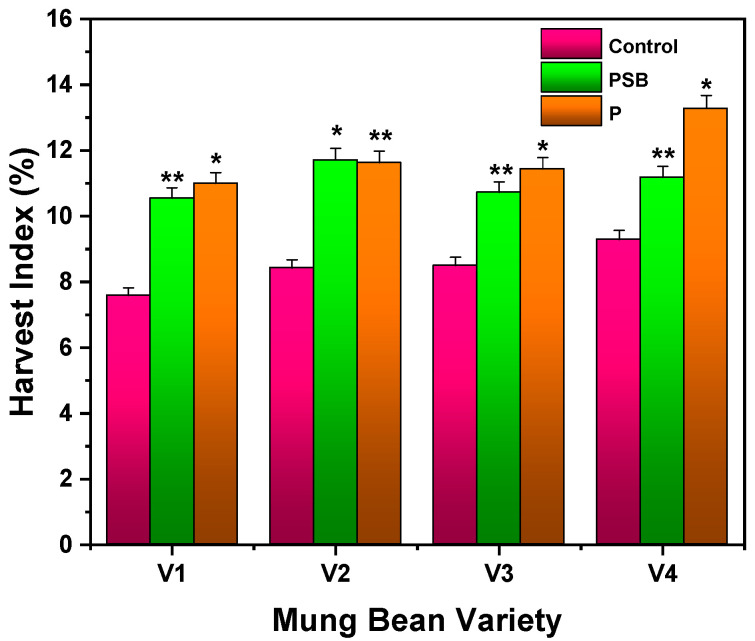
Harvest index of different varieties of mung bean in inoculation with PSB (*Pseudomonas* spp.) and P after 60 days of observation. Each treatment was the mean of three replicates. The upper bar represents the standard error of the means (mean ± SEM). * Represents *p* ≤ 0.01 and ** represents *p* ≤ 0.05 of results.

Figure 5 shows the results of the harvest index of inoculated varieties of mung bean with P and PSB. It was observed that V4-P-treated mung bean showed a better harvest index followed by PSB than the control (*p* ≤ 0.01). The maximum harvest index of about 13.28% (V4) was observed with P and 11.71% (V2) with PSB. Ebbisa and Amdemariam [30] reported that faba bean plants showed the highest harvest index of 59.91% by applying biofertilizer of farmyard manure with inoculation of *Rhizobium* at 100% rate of NPS, while the lowest harvest index of 39.37% was recorded by applying biofertilizer of farmyard manure with *Rhizobium* inoculation at 0% a rate of NPS. Chattha et al. [27] revealed that a maximum harvest index of 0.213% was achieved in mung bean plants by treating with *Rhizobium* and PSB.

### 2.3. Physiological Parameters

Physiological parameters discussed the photosynthetic yield (PSY), photosynthetic active radiation (PSAR), electron transport rate (ETR), and momentary fluorescent (MF) rate of four varieties of mung bean after administration of PSB and P-alone. Table 2 reveals that V1, V2, V3, and V4 varieties of mung bean treated with PSB (100 g/kg seed) showed the maximum photosynthetic yield of 0.69, 0.70, 0.68, and 0.75 μmol/m^2^ s, respectively. Similarly, the blend-P-treated mung bean presented the highest photosynthetic yield of about 0.63, 0.68, 0.69, and 0.68 μmol/m^2^ s for V1, V2, V3, and V4, respectively. Analyzed data regarding photosynthetic yield indicated the significance of results among interactions of varieties, phosphorus, and PSB by showing a maximum photosynthetic yield of 0.75 μmol/m^2^ s in PSB-treated (100 g/kg seed) V4 variety of mung bean (see Table 2). It was also observed that photosynthetic active radiation (PSAR), electron transport rate (ETR), and momentary fluorescent (MF) rate were noted as 297.65, 79.73, and 360.33 μmol/m^2^ s for V1, 364.32, 87.72, and 365.33 μmol/m^2^ s for V2, 293.42, 83.21, and 337.22 μmol/m^2^ s for V3, and 302.14, 96.12, and 355.33 μmol/m^2^ s for V4 variety of mung bean treated with PSB. Based on the results, it was noted that the maximum PSAR and MF observed in the V2 variety of mung bean treated with PSB, while maximum ETR was observed in the V4 variety of mung bean treated with PSB. Overall, it was indicated that PSB-treated mung bean presented the best results as compared to bland P-treated mung bean and control.

### 2.4. Soil Parameters Analysis

**Figure 6:**
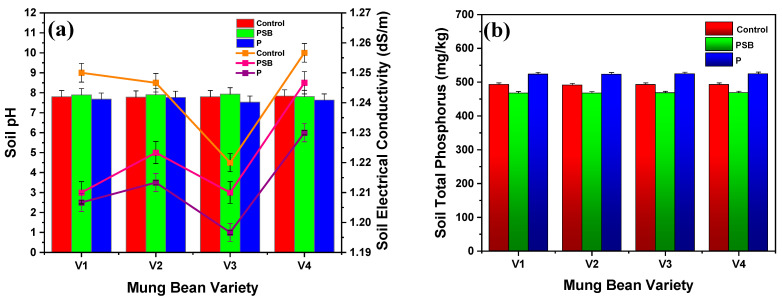
Comparative results of (**a**) soil pH and EC, and (**b**) soil total phosphorus content of different varieties of mung bean with inoculation with PSB and P after 60 days. Each treatment was the mean of three replicates. The upper bar represents the standard error of the means (mean ± SEM).

Soil pH, soil electrical conductivity (EC), and total soil phosphorus were examined after harvesting of the mung bean crop. Figure 6a,b presents the results of soil pH, soil electrical conductivity (EC), and total soil phosphorus for four varieties of mung bean after 60 days of assessment. Results showed that after applying P-treated mung bean, the soil pH and soil EC tend to decrease a little bit, but with PSB the soil pH remains constant to the control (pH 7.00). The control showed the maximum soil EC of 1.25 dS/m for V1, 1.24 dS/m for V2, 1.22 dS/m for V3, and 1.25 dS/m for V4, while PSB- and P-inoculated mung bean presented the maximum EC of 1.21 and 1.20 dS/m for V1, 1.22 and 1.21 dS/m for V2, 1.21 and 1.19 dS/m for V3, and 1.24 and 1.23 dS/m for V4, respectively. Results also revealed a very small difference between control and PSB- and P-treated mung bean regarding soil pH and soil EC. Figure 6b shows the results of total soil P for all four varieties of mung bean. Total soil P is comprised of available soil P, and organically and inorganically bound soil phosphorus. The recorded data of soil phosphorus indicated that the V1 variety possesses the highest phosphorus in the soil of about 492.89 mg/kg with control, 467.69 mg/kg with PSB, and 523.67 mg/kg with P-alone. It could be observed that there was a very small difference in all varieties of mung beans. Overall, it is concluded that the V1 variety of mung bean showed the best results with P-alone, which indicated that direct use of phosphorus in plants has a significant increase in soil phosphorus rather than PSB. Parastesh et al. [22] revealed that 30 days of inoculation of vermicompost with bacterial strains significantly increased the total soil P content from 10 to 357 mg/kg and also application of vermicompost with bacterial strains (T2) effectively reduced the soil pH to 7.6 from an initial pH of 8.4. Hassan et al. [17] reported that PSB caused increased phosphorus content in soil by using PSB (*Bacillus polymyxa*) of 13.50 mg/kg and PSB (*Pseudomonas*) of 11.50 mg/kg, while the control shows the lowest phosphorus content in soil of 3.5 mg/kg. Rafique et al. [25] stated that co-inoculation of maize plant seed with PSB (*L. fusiformis*) and bagasse biochar showed the nitrogen (N), phosphorus (P), and potassium (K) concentration of 10.24 ± 0.51, 17.19 ± 0.68, and 166.25 ± 5.62 mg/kg soil, respectively, after 45 days of harvesting.

### 2.5. Mechanism of P Solubilization by PSB

**Figure 7:**
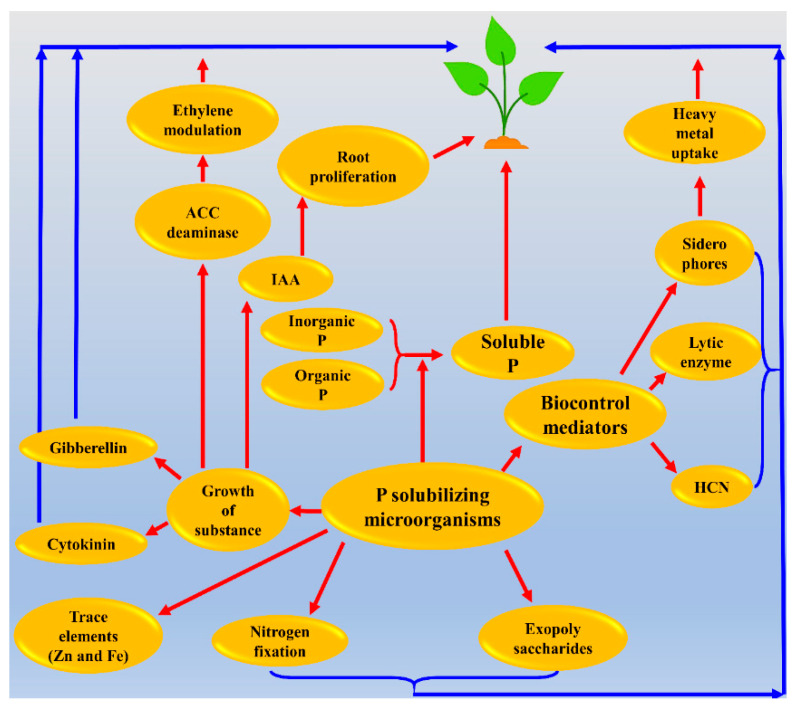
Mechanisms of phosphate solubilization and plant growth promotion by phosphorus-solubilizing bacteria. This figure is reproduced from Sharma et al. [35].

It is widely shown that organic acids synthesized by soil microorganisms are the primary mechanism of mineral phosphate solubilization (see Figure 7) [31,32]. Organic acids released from bacteria are responsible for acidifying the microbial cell and its surrounding environment. Subsequently, P may be released by proton substitution for Ca^2^^+^ from a mineral phosphate. Organic phosphate soluble acids, which are formed during the phosphate solubilization process, play a very important role. The most common solvency agent in mineral phosphate is gluconic acid. Besides gluconic acid, several other organic acids including malonic, glycolic, oxalic, and succinic acid have been identified among phosphate solubilizers [33]. Organic acids play a major role in the solubilization of mineral phosphate backed by experimental evidence. The amount of P that was solubilized by the whole culture was nearly equal to the solubilizing ability of organic acids, isolated from a culture of *Rhizobium leguminosarum* [34]. Other processes, such as the development of inorganic, sulfuric [35], nitric, and carbonic acids [36], and microorganisms chelating substances [37] have been proposed. However, the role of these mechanisms in phosphorus release seems to be insignificant and their efficiency has still not been understood.

## 3. Materials and Methods

### 3.1. Soil Sampling, Characteristics, and Field Layout Plan

Prior to this research, for physico-chemical analysis, the soil samples of different properties were collected from the experimental trial site with a depth of 0–30 cm with sandy clay loam (58.2% sand, 30.1% silt, and 12.05% clay) texture possessing (pH = 7.89, EC = 1.27 dS/m) phosphorus 100 mg/kg, potassium 128.32 ppm, nitrogen 0.50%, and organic matter 0.62 (see Table 3). Three channels were prepared with the length of 12 m and a width of 27 m, while the main water channel was 1.5 m, subwater channel was 1.5 m, and subpath in each channel was 1.0 m. Sixteen samples were sown in each channel, namely, (T_1_, T_2_, T_3_, …, T_16_) [38,39]. Due to annual rainfall of 300 mm, Faisalabad climatically falls in the semi-arid and subtropical category. The crop was sown in July and was harvested after 120 days. During this period, the prevailing noted climate conditions are mentioned below in Table 4. According to the data, the average temperature range was recorded as 31–33 °C, humidity 70–75%, and a minimum and maximum rainfall of 5.4 and 26.6 mm, respectively.

### 3.2. Experimental Design

The randomized complete block design (RCBD) having a factorial arrangement was performed as an experimental trial with three replications that comprised two inoculation levels of phosphorus-solubilizing bacteria (PSB) *Pseudomonas* spp., which was collected from the microbiological section of the Ayub Agriculture Research Institute Faisalabad, Pakistan (B_1_ = control, B_2_ = 100 g/kg seed) along with phosphorus (P = 70 kg/ha) by testing on four different varieties of mung bean including V_1_ = PRI-2018, V_2_ = NM-16, V_3_ = NM-11, V_4_ = AZRI-06 after 60 days, obtained from the Nuclear Institute for Agriculture and Biology (NIAB), Faisalabad, Pakistan and Pulses Research Institute, Ayub Agriculture Research Institute, Faisalabad, Pakistan, while nitrogen, phosphorus, and potassium fertilizers were applied as urea, SSP, and MOP at the rate of 30, 70, and 50 kg/ha, respectively [27].

### 3.3. Preparation and Application of Inoculum

Inoculation was carried out with the recommended dose of inoculum (100 g/kg seed) in which a 10% glucose solution was prepared at the ratio of 10 g/100 mL. For each experimental unit, the inoculum slurry was prepared separately by mixing up 2.5 g of PSB inoculum material with the measured amount of glucose solution. By mixing the seed with slurry, the seed of each mung bean variety was inoculated until the seed was fully covered with the slurry. Then the seed was shade dried for 12 h after which it was inoculated and ready for sowing [40].

### 3.4. Soil Management and Crop Husbandry

The seed of each genotype of mung bean was sown in each plot at a 1 cm depth of soil. For inoculated treatment, seeds of the four varieties were inoculated before sowing using seed at the rate of 30 kg/ha. For seedbed preparations, the soil was well pulverized by two cultivations and followed by planking. The single-row drill (Millat, Pakistan) was used for sowing to maintain a 30 cm distance between the lines. To maintain a 10 cm distance between plants, thinning was performed ten days after sowing. The application of fertilizer was performed using the sources of urea, SSP, and MOP with the dose of NPK at the rate of 30, 70, and 50 kg/ha, respectively. The agronomic practices for all the treatments were maintained for their uniformity. The crop was protected from different insects, weeds, and diseases by adopting proper crop days when pods were fully ripened, and then threshing was conducted after sun drying.

### 3.5. Measurements

#### 3.5.1. Agronomic Parameters

After the emergence of the first seedling in the plot, the number of visible seedlings was counted daily. Emergence percentage of agronomical parameters was restrained with a meter rod and measuring balance by taking an average of various parameters including shoot length (cm), root length (cm), shoot fresh weight (g), root fresh weight (g), shoot dry weight (g), root dry weight (g), root and shoot phosphorus contents, number of plant leaves, leaf area (cm^2^), leaf area index, and biological yield (kg/ha) [41].

#### 3.5.2. Physiological and Yield-Related Parameters

These parameters were recorded using a photosynthetic yield analyzer (Mini-Pam-II, Pfullingen, Germany) consisting of photosynthetic yield (μmol/m^2^ s), photosynthetically active radiation (μmol/m^2^ s), electron transport rate (μmol/m^2^ s), and momentary fluorescent rate (μmol/m^2^ s). Yield-related parameters such as number of grains per pod, number of pods per plant, number of pod-bearing branches, grain yield (kg/ha), and 1000-grain weight (g) were also measured. Similarly, the harvest index (%) was measured by following Equation (1) [42].

(1) $$\mathrm{HI}\;\left(\%\right)=\frac{Economical\;yield}{Biological\;yield\;}\times 100$$

#### 3.5.3. Soil Parameters

Plant P content (mg/plant) was evaluated in shoot and root by the digestion of samples. Prior to digestion, 0.25 g of oven-dried and ground plant material was placed into a 100 mL flask. Then, a 2.5 mL mixture of HNO_3_ and HClO_3_ with a ratio of 2:1 was added into this flask and covered with aluminum foil for 24 h. Then, aluminum foil was removed from its opening and the flask was placed on a hot plate and the temperature was slowly increased to 235 °C for 1.5 h until the dense white fumes were produced. The digested liquid was filtered through filter paper (Whatman No. 1). Then, 1 mL of clear filtrate was taken off along with 1 mL mixture of ammonium heptamolybdate ((NH_4_)_6_Mo_7_O_24_) and ammonium metavanadate (NH_4_VO_3_) reagent was added into a 10 mL test tube and made its final volume up to 10 mL by adding d.H_2_O. A series of standard solutions (0.5, 1, 1.5, 2, and 2.5 ppm) were made by diluting the stock solution (KH_2_PO_4_). After that, readings were recorded from a UV-vis spectrophotometer (V-73, Japan) at the wavelength of 410 nm. Further calculations were processed by the following Equation (2) [43,44].

(2) $$P\;\left(ppm\right)=ppm\;P\;\left(from\;calibration\;curve\right)\frac{V}{Wt}\times \frac{V2}{V1}\;$$

where *V* is the final volume of plant/soil digested material after dilution (mL), *V*_1_ is the volume of plant/soil digest used for *P* assessment purposes (mL), *V*_2_ is the volume of flask used for assessment (mL), and *Wt* is the dry weight of plant/soil (g) [43].

### 3.6. Statistical Analysis

Data of each parameter were taken in three replicates and shown as means ± standard error of the means (mean ± SEM). The significance of variables was assessed by analysis of variance (ANOVA) and means were ranked on Tukey’s HSD (honestly significant difference) test (*p* ≤ 0.05) using Minitab^®^ Statistical Software (version: 20.3, USA) [44].

## 4. Conclusions

The present study explained the effect of inoculation of phosphorus-solubilizing bacteria (PSB; *Pseudomonas* spp.) and phosphorus (P) on four different varieties of mung bean to increase its cultivation and gain yield. The general conclusion of the present study is that PSB inoculation of mung bean genotypes resulted in increased seed yield, 1000-grain weight, and biological yield. It was also revealed that PSB inoculation increased the shoot and root P concentration and uptake in mung bean varieties. Based on the results, the farmer could use PSB to enhance mung bean yield with the help of PSB inoculation even with less P fertilization. However, it is recommended that more study is needed on different soil types to confirm the effects of PSB (*Pseudomonas* spp.) on mung beans. Moreover, the inoculation of PSB with different crop types is required in the near future to expand its use in different crop cultivations.

## Figures and Tables

**Table 1:** The number of leaves, leaf area, and leaf area index of four varieties of mung bean affected by PSB and P inoculation (mean ± SEM).

Mung Bean Variety	Number of Leaves (per Plant)			Leaf Area (cm^−2^)			Leaf Area Index (cm)		
	Control	PSB	P	Control	PSB	P	Control	PSB	P
V1	18 ± 0.36	21 ± 0.42	18 ± 0.36	582.22 ± 11.64	743.55 ± 14.87	628.88 ± 12.57	1.86 ± 0.037	2.14 ± 0.042	1.96 ± 0.039
V2	16 ± 0.32	17 ± 0.34	18 ± 0.36	474.77 ± 9.49	508.11 ± 10.16	622.77 ± 12.45	1.51 ± 0.030	1.60 ± 0.033	1.78 ± 0.035
V3	16 ± 0.32	17 ± 0.34	16 ± 0.32	486.31 ± 9.72	546.44 ± 10.92	628.55 ± 12.57	1.59 ± 0.031	1.69 ± 0.033	1.81 ± 0.036
V4	17 ± 0.34	22 ± 0.44	19 ± 0.38	461.66 ± 9.23	563.88 ± 11.27	522.33 ± 10.44	1.58 ± 0.031	1.66 ± 0.033	1.59 ± 0.032

V1: PRI-2018, V2: NM-16, V3: NM-11, and V4: AZRI-06. PSB: phosphorus-solubilizing bacteria (100 g/kg seed) and P: phosphorus (70 kg/ha). Each treatment was the mean of three replicates.

**Table 2:** Momentary fluorescent rate, photosynthetic active radiation, photosynthetic yield, and electron transport rate of mung beans as affected by different levels of PSB and P inoculation (mean ± SEM).

Parameter	Mung Bean Variety (μmol/m^2^ s)			
	V1	V2	V3	V4
Momentary fluorescent rate (MF)				
Control	353.21 ± 7.06	296.13 ± 5.92	298.35 ± 5.96	348.66 ± 6.97
PSB	360.33 ± 7.20	365.33 ± 7.31	337.22 ± 6.74	355.33 ± 7.10
P	378.19 ± 7.56	400.53 ± 8.01	301.33 ± 6.03	320.27 ± 6.40
Photosynthetic active radiation (PSAR)				
Control	293.33 ± 5.86	326.66 ± 6.53	325.33 ± 6.50	290.66 ± 5.81
PSB	297.65 ± 5.93	364.32 ± 7.28	293.42 ± 5.86	302.14 ± 6.04
P	337.54 ± 6.75	305.32 ± 6.10	336.67 ± 6.73	291.33 ± 5.83
Photosynthetic yield (PSY)				
Control	0.69 ± 0.014	0.68 ± 0.014	0.67 ± 0.013	0.67 ± 0.013
PSB	0.69 ± 0.014	0.70 ± 0.014	0.68 ± 0.013	0.75 ± 0.015
P	0.63 ± 0.012	0.68 ± 0.013	0.69 ± 0.014	0.68 ± 0.013
Electron transport rate (ETR)				
Control	84.32 ± 1.68	90.21 ± 1.80	89.36 ± 1.78	84.93 ± 1.69
PSB	79.73 ± 1.59	87.72 ± 1.75	83.21 ± 1.66	96.12 ± 1.92
P	90.16 ± 1.80	90.86 ± 1.81	99.24 ± 1.98	95.36 ± 1.90

V1: PRI-2018, V2: NM-16, V3: NM-11, and V4: AZRI-06. PSB: phosphorus-solubilizing bacteria (100 g/kg seed) and P: phosphorus (70 kg/ha). Each treatment was the mean with three replicates.

**Table 3:** Physico-chemical properties of composite soil (mean ± SEM).

Parameter	Concentration
Texture	Sandy loam
Sand (%)	58.20 ± 0.62
Silt (%)	30.10 ± 0.81
Clay (%)	12.05 ± 0.31
pH	7.89 ± 0.05
Electrical conductivity (dS/m)	1.27 ± 0.05
Phosphorus (mg/kg)	8.65 ± 0.22
Potassium (mg/L)	128.32 ± 1.96
Total nitrogen (%)	0.050 ± 0.001
Organic matter (%)	0.62 ± 0.025

Each factor was measured with three replicates.

**Table 4:** Weather data during the autumn season of mung bean sowing (mean ± SEM).

Month	Week	Average Temperature (°C)	Mean Relative Humidity (%)	Total Rainfall (mm)
July	22 Jul–28 Jul	30.28 ± 1.51	71.42 ± 3.57	17.61 ± 0.88
	29 Jul–4 Aug	32.71 ± 1.63	71.28 ± 3.56	0.60 ± 0.03
August	5 Aug–11 Aug	32.70 ± 1.64	78.85 ± 3.94	65.62 ± 3.28
	12 Aug–18 Aug	33.22 ± 1.66	73.85 ± 3.69	11.00 ± 0.55
	19 Aug–25 Aug	33.44 ± 1.67	67.00 ± 3.35	3.41 ± 0.17
	26 Aug–01 Sep	34.34 ± 1.72	69.28 ± 3.46	0.90 ± 0.04
September	02 Sep–08 Sep	33.65 ± 1.68	65.71 ± 3.28	---
	09 Sep–15 Sep	34.80 ± 1.74	67.00 ± 3.35	---
	16 Sep–22 Sep	32.28 ± 1.61	72.71 ± 3.63	6.63 ± 0.33
	23 Sep–29 Sep	31.05 ± 1.55	73.42 ± 3.67	15.20 ± 0.76
October	30 Sep–06 Oct	27.18 ± 1.35	76.92 ± 3.84	1.91 ± 0.09
	07 Oct–13 Oct	26.72 ± 1.33	62.07 ± 3.10	6.41 ± 0.32
	14 Oct–20 Oct	25.61 ± 1.28	69.21 ± 3.46	30.30 ± 1.51
	21 Oct–27 Oct	24.47 ± 1.22	73.85 ± 3.69	---
November	28 Oct–03 Nov	24.05 ± 1.20	77.00 ± 3.85	1.34 ± 0.067
	04 Nov–10 Nov	20.74 ± 1.04	72.01 ± 3.60	---

Note: Each treatment was the mean of three replicates.

## Data Availability

Not applicable.
